# A chimeric vaccine protects farmed saltwater crocodiles from West Nile virus-induced skin lesions

**DOI:** 10.1038/s41541-023-00688-w

**Published:** 2023-06-27

**Authors:** Gervais Habarugira, Jessica J. Harrison, Jasmin Moran, Willy W. Suen, Agathe M. G. Colmant, Jody Hobson-Peters, Sally R. Isberg, Helle Bielefeldt-Ohmann, Roy A. Hall

**Affiliations:** 1 School of Veterinary Science, The University of Queensland, Gatton, QLD Australia; 2 School of Chemistry and Molecular Biosciences, The University of Queensland, St Lucia, Australia; 3 Centre for Crocodile Research, Noonamah, NT Australia; 4 Australian Infectious Diseases Research Centre, The University of Queensland, St Lucia, QLD Australia; 5 Present Address: Australian Centre for Disease Preparedness, The Commonwealth Scientific and Industrial Research Organisation, Geelong, VIC 3219 Australia; 6 Present Address: Unité des Virus Émergents (UVE) Aix-Marseille Univ-IRD 190-Inserm 1207, Marseille, France

**Keywords:** West nile virus, Live attenuated vaccines

## Abstract

West Nile virus (WNV) causes skin lesions in farmed crocodiles leading to the depreciation of the value of their hides and significant economic losses. However, there is no commercially available vaccine designed for use in crocodilians against WNV. We tested chimeric virus vaccines composed of the non-structural genes of the insect-specific flavivirus Binjari virus (BinJV) and genes encoding the structural proteins of WNV. The BinJV/WNV chimera, is antigenically similar to wild-type WNV but replication-defective in vertebrates. Intramuscular injection of two doses of BinJV/WNV in hatchling saltwater crocodiles (*Crocodylus porosus*) elicited a robust neutralising antibody response and conferred protection against viremia and skin lesions after challenge with WNV. In contrast, mock-vaccinated crocodiles became viraemic and 22.2% exhibited WNV-induced lesions. This suggests that the BinJV/WNV chimera is a safe and efficacious vaccine for preventing WNV-induced skin lesions in farmed crocodilians.

## Introduction

West Nile virus (WNV), including the Australian Kunjin strain (WNV_KUN_), is a mosquito-borne zoonotic virus of global health concern and has been responsible for several outbreaks in a range of species, including birds, horses, humans and reptiles. The virus transmission cycle is maintained between birds and mosquitoes, primarily mosquitoes of the Culex spp.^1–5^. However, additional modes of transmission, such as fecal-oral transmission, have been reported in crocodilians^6–8^.

With some notable exceptions (e.g. corvids), infected mammals and birds generally develop a subclinical disease characterised by non-specific fever^9^. A small proportion of infected individuals amongst humans, sheep, horses, birds and alligators develop a severe meningoencephalitis syndrome^10–14^. The clinical picture in crocodilians varies widely from subclinical infection in saltwater crocodiles^15^ to severe disease in American alligators (*Alligator mississippiensis*) characterised by neurological and digestive tract syndromes^8^. They also develop skin lesions known as 'pix'^8,16^. While infected saltwater crocodiles (*Crocodylus porosus*) do not exhibit overt clinical disease, they do develop 'pix' skin lesions that cause depreciation and rejection of the hides from farmed animals during their processing for leather^15^. Hide rejections result in large economic losses, which threaten industry viability and the sustainable use conservation programme that employs indigenous Australians and has returned this apex predator from the brink of extinction^17^. Infected saltwater crocodiles and alligators can also develop viraemic titres sufficiently high to sustain a transmission cycle with feeding mosquitoes^6,7^. Given this potential role as an amplifying host, protecting farmed saltwater crocodiles from WNV infection would not only reduce industry losses but also serve, by extension, to protect humans and other animal hosts.

Currently, there are four WNV vaccines approved for veterinary use in the USA for horses. Most of these vaccines must be administered biannually to be fully protective^18^. One inactivated vaccine (West Nile Virus Vaccine, Boehringer Ingelheim Vetmedica, Inc. [BIVI], St. Joseph, MO), commercially available for use in horses, has been used off-label in alligators^18,19^. A subsequent report questioned vaccine safety since WNV viral genomic material was detected in tissue samples from vaccinated alligators. However, it is not yet clear at this stage whether the detected viral RNA was from the vaccine rather than a natural infection^20^. Regardless, none of these vaccines are licensed and available in Australia; hence there is a need for a vaccine for use in farmed saltwater crocodiles, an important industry in Northern Australia.

In the quest for reliable and safe vaccine candidates against pathogenic flaviviruses, a chimeric vaccine platform was developed based on an insect-specific flavivirus (ISF)^21^. The platform consists of exchanging the structural proteins prM and envelope (E) of a newly discovered ISF, known as Binjari virus (BinJV), with those of pathogenic flaviviruses^21^. The resulting chimeric virions are structurally and antigenically similar to parental pathogenic flaviviruses but retain the ISF phenotype and cannot replicate in vertebrate cells, rendering them safe vaccine candidates in vertebrates^21,22^. Herein we report on our investigations of the efficacy of BinJV/WNV as a vaccine candidate to protect saltwater crocodiles from WNV infection and the development of pix lesions.

## Results

Since there are currently no commercial WNV vaccines available in Australia, we investigated the immunogenicity and safety of two chimeric vaccine candidates in saltwater crocodile hatchlings devoid of maternal antibodies. BinJV/WNV_KUNproto_ contains the prM-E sequence from the 1960 prototype isolate of WNV_KUN_, while BinJV/WNV_KUN2011_ contains the prM-E sequence from a 2011 isolate of WNV_KUN_.

### Vaccine immunogenicity

**Fig. 1:**
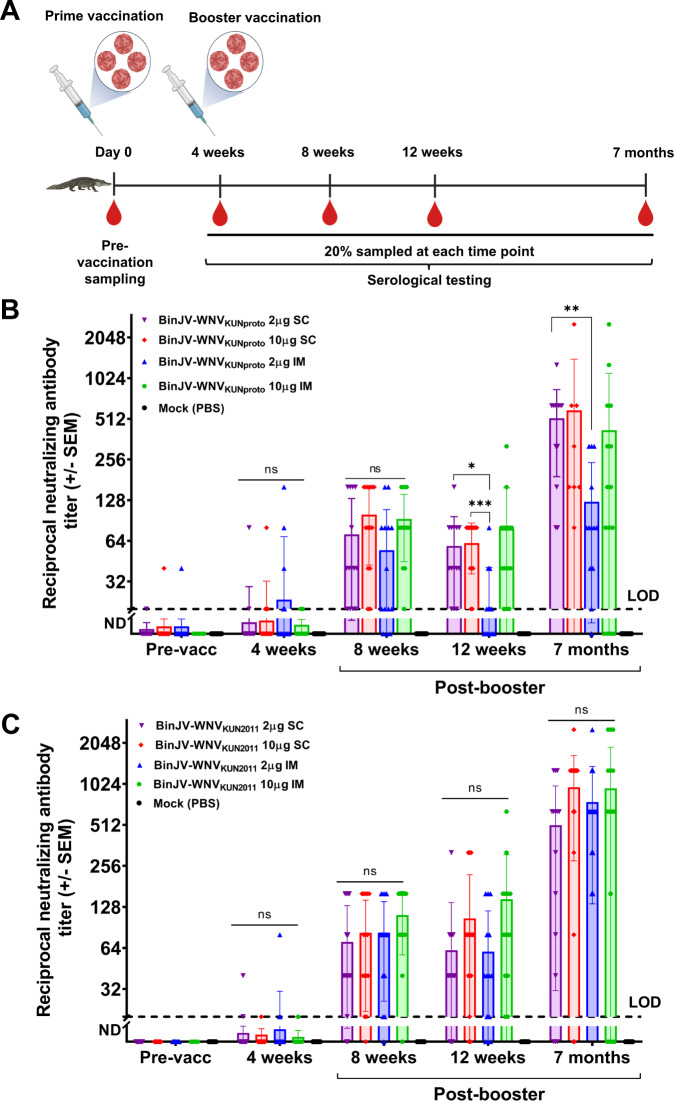
Vaccination schedules and virus neutralisation antibody titres in saltwater crocodiles vaccinated with two vaccine candidates at multiple time points (pre-vaccination, 4 weeks, 8 weeks and 7 months post-vaccination) via subcutaneous (SC) or intramuscular (IM) routes. **A** Schedule of vaccination and sampling for the immunogenicity study of chimeric BinJV/WNV_KUN_ vaccines. Four months old crocodile hatchling free of anti-WNV_KUN_ maternal antibodies received two doses of chimeric vaccine at 4 weeks interval, by either the subcutaneous or intramuscular route. Samples were tested in VNT for seroconversion. **B** Neutralising antibody titres in animals vaccinated with 2 or 10 µg of BinJV/WNV_KUNproto_ either SC or IM. **C** Neutralising antibodies titres in crocodiles vaccinated with BinJV/WNV_KUN2011_. The dotted line represents the lower limit of detection (titre of 20) of virus-neutralising antibodies by VNT. The upper limit of detection of the assay was ≥2560. The two-way ANOVA with Tukey’s post-test statistical analysis to test differences in virus-neutralising antibody titres between treatment groups at various time points. Significant statistical difference thresholds are *p ≤ 0.05, **p ≤ 0.01 and ***p ≤ 0.001.

Groups of 70 crocodile hatchlings were immunised subcutaneously (SC) or intramuscularly (IM), with two doses of each vaccine candidate as per Table 1 and 20% of animals in each group were sampled at time points indicated in Fig. 1A.

**Table 1:** Doses and routes of administration of vaccine candidates used in immunogenicity and safety study.

Treatment group/Vaccine	Type	Dose	Route	Number of immunisations	Number of vaccinated animals
BinJV/WNV_KUNproto_	Chimeric vaccine	10 µg	IM	2	70
		10 µg	SC	2	70
		2 µg	IM	2	70
		2 µg	SC	2	70
BinJV/WNV_KUN2011_	Chimeric vaccine	10 µg	IM	2	70
		10 µg	SC	2	70
		2 µg	IM	2	70
		2 µg	SC	2	70
Mock vaccinated	PBS	-	IM	2	140

By four weeks post prime (after the first vaccination) (pre-booster in Fig. 1B, C), only 14 to 35% (2/14– 5/14) of crocodiles in each vaccination group had developed detectable neutralising antibodies to the WNV_KUNV2011_ strain. The maximum titres ranged from 20 to 160. However, by 8–12 weeks post prime (4–8 weeks post booster), most (79–100%) of the animals in each vaccination group had seroconverted. The median neutralising antibody titre for the vaccinated groups ranged from 10 to 120, (with individual titres ranging from <20 to 640) (Table 2, Supplementary Table 8 and Supplementary Fig. 3). Notably, the neutralising titres had risen by 7 months post prime vaccination (6 months post booster) in all groups with median neutralising antibody titres ranging from 80 to 1280 (individual range of <20–≥2560) (Table 2, Supplementary Table 9 and Supplementary Fig. 3), suggesting a relatively delayed peak of neutralising antibody response in crocodiles (Fig. 1B, C). While there was no statistically significant difference between the various groups vaccinated with BinJV/WNV_KUN2011_ at any time point (Fig. 1C), there were differences between some of the BinJV/WNV_KUNproto_ groups. The neutralising titres were statistically different at 12 weeks (*p = 0.0179) and 7 months post-vaccination (**p = 0.0074) between groups receiving 2 µg SC and 2 µg IM of BinJV/WNV_KUNproto_, and at 12 weeks post prime (8 weeks post booster) between the 2 µg IM and 10 µg SC groups (***p = 0.0007) receiving the same vaccine (Fig. 1B). There was no statistically significant difference in neutralising antibody titres between BinJV/WNV_KUNproto_ and BinJV/WNV_KUN2011_ vaccination groups at 12 weeks post prime (8 weeks post booster) irrespective of vaccine dose and route of administration (*p* ≤ 0.5), although slightly lower titres were observed at these time points in animals that received BinJV/WNV_KUNproto_ by IM route (Supplementary Fig. 3 and Table 1). By 7 months post prime, neutralising antibody titres also tended to be lower in groups having received BinJV/WNV_KUNproto_, with titres statistically lower for 2 µg of BinJV/WNV_KUNproto_ administered via IM route (titre of 80) compared to 2 µg of BinJV/WNV_KUN2011_ by the same route (titre of 640) (*p = 0.0348) or compared to 10 µg of BinJV/WNV_KUN2011_ administered through subcutaneous route (titre of 1280) (**p = 0.0057). Neither BinJV/WNV_KUN2011_ nor BinJV/WNV_KUNproto_ caused any noticeable side effects in vaccinated crocodiles, irrespective of vaccine dosage or route of administration.

**Table 2:** Comparison of median neutralising antibody titres induced by BinJV/WNV_KUNproto_ and BinJV/WNV_KUN2011_.

Time point (Post-prime)	2 μg SC		2 μg IM		10 μg SC		10 μg IM		Mock (PBS)
	B/WNV_KUNproto_	B/WNV_KUN2011_	B/WNV_KUNproto_	B/WNV_KUN2011_	B/WNV_KUNproto_	B/WNV_KUN2011_	B/WNV_KUNproto_	B/WNV_KUN2011_	
Pre-vaccination	<20	<20	<20	<20	<20	<20	<20	<20	<20
4 weeks	<20	<20	<20	<20	<20	<20	<20	<20	<20
8 weeks	40	40	30	80	80	80	80	120	<20
12 weeks	40	40	10	40	80	80	60	80	<20
7 months	640	640	80	640	240	1280	160	640	<20

### Vaccine safety: no evidence of BinJV/WNV_KUN_ replication in crocodiles

To confirm that the insect-specific host restriction of the vaccine candidates extended to crocodilian species in vivo, we investigated whether the BinJV/WNV_KUN_ chimeric viruses could replicate in vaccinated crocodiles. Results using BinJV NS5- and NS3-specific RT-PCR and qRT-PCR on plasma and cloacal swabs from vaccinated crocodiles and pen water samples were all negative. The lack of detectable viral RNA in all tested samples using these sensitive assays suggested that the chimeric viruses did not replicate, or that replication was below the limit of detection, in crocodiles vaccinated with BinJV/WNV_KUN_ (Supplementary Fig. 1). This finding corroborates the inability of the chimeric vaccine to replicate in saltwater crocodile-derived cell lines^21^.

### WNV_KUN_ challenge of crocodiles vaccinated with BinJV/WNV_KUN_

The BinJV/WNV_KUN2011_ vaccine was selected for use in a subsequent challenge study to test for protection efficacy against WNV infection and skin lesions. This selection was based on its slightly better performance in the immunogenicity study above, the higher yields it gave in production^21^, and our previous findings that all WNV_KUN_ RNA detected in lesions of naturally infected crocodiles was genetically more similar to WNV_KUN2011_. The selected chimeric vaccine is henceforth referred to simply as BinJV/WNV_KUN_ for clarity.

This experiment used a 10 µg dose of the vaccine formulated with or without an adjuvant (Advax™) administered intramuscularly (IM). We also assessed the effect of UV-C inactivation of the vaccine on immunogenicity and efficacy to address the possibility that low but undetectable levels of replication of the vaccine in crocodiles enhanced the immune response.

As observed in the immunogenicity study above, low levels of neutralising antibodies (titres of 20–40) were first detected at 4 weeks post prime immunisation in a few crocodiles in all treatment groups, with the exception of the non-adjuvanted inactivated vaccine group (Supplementary Tables 9 and Supplementary Fig. 4). By 8 weeks post prime vaccination (pre-challenge), all animals (100%, *n* = 25) vaccinated with live-adjuvanted BinJV/WNV_KUN_ had neutralising antibodies while 23 of 25 (92%) animals vaccinated with live non-adjuvanted vaccine had neutralising antibodies. At the same time point, 24 of 25 (96%) animals immunised with inactivated adjuvanted BinJV/WNV_KUN_ had neutralising antibodies, while 18 of 25 (72%) animals vaccinated with inactivated non-adjuvanted BinJV/WNV_KUN_ had neutralising antibodies. The median neutralising antibody titre at pre-challenge sampling was 160 (range <40–≥2560) for the live-adjuvanted vaccine, 80 (range <20–1280) for the live non-adjuvanted, 40 (range <20–640) for the inactivated adjuvanted vaccine and 20 (range <20–320) for inactivated non-adjuvanted vaccine (Fig. 1C and Supplementary Fig. 4). While there was an apparent trend of enhanced immunogenicity between vaccination groups (live-adjuvanted > live-unadjuvanted > inactivated adjuvanted > inactivated unadjuvanted), virus-neutralising antibody titres were not statistically different between treatment groups at four weeks post prime vaccination (pre-booster) or four weeks post booster (pre challenge) (Supplementary Fig. 4). While neutralising antibody titres had increased by 5 weeks post-challenge (13 weeks post prime vaccination) relative to pre-challenge levels, the increase was not statistically different between these time points.

### BinJV/WNV_KUN_ vaccine protects crocodiles against viraemia and the development of 'pix' lesions

None of the vaccinated animals from the four vaccine groups were viraemic as assessed by qRT-PCR 4 days post-challenge, while four of ten randomly selected animals from the mock vaccinated group tested positive for WNV_KUN_ by qRT-PCR (Supplementary Table 4). Twenty-one days post-challenge, 23 of 27 (85.2%) animals from the mock-vaccinated group had developed neutralising antibodies confirming a productive infection in most animals after the virus challenge.

**Fig. 2:**
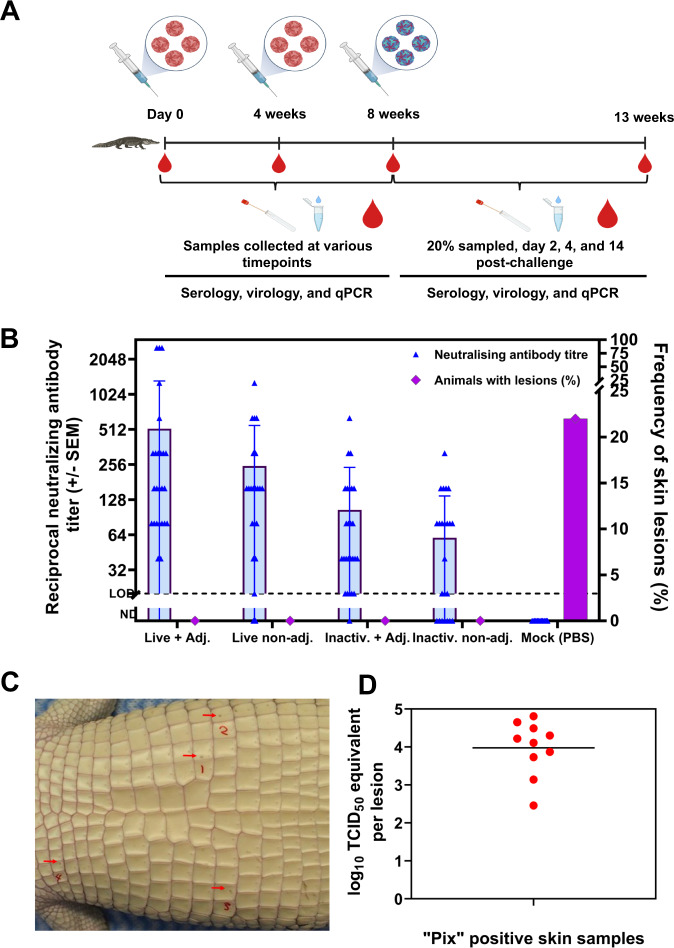
Serology and pathology in vaccinated and mock-vaccinated saltwater crocodiles. **A** Four-month-old crocodile hatchlings free of anti-WNV_KUN_ maternal antibodies received two doses of BinJV/WNV_KUN_ vaccine intramuscularly at a 4-week interval. Four weeks after the booster vaccination, the hatchlings were challenged with 10^5^ TCID_50_ of WNV_KUN_. Samples (blood, cloacal swabs, and water) were collected at various time points. **B** Comparison of neutralising antibody titre at 8 weeks post-vaccination (pre-challenge) and animals with pix skin lesions among treatment groups. The dotted line represents the lower limit of detection (titre of 20) of virus-neutralising antibodies by VNT. The dotted line in panels **B** and **C** represents the limit of detection. The upper limit of detection of the assay was ≥2560. **C** Macroscopic appearance of pix skin lesions developed in mock vaccinated crocodiles (red arrows). **D** Virus titre TCID_50_ equivalent per lesions detected and quantified by WNV_KUN_ qRT-PCR in pix skin lesions from mock-vaccinated challenged animals.

None of the vaccinated crocodiles developed 'pix' skin lesions. In contrast, six of 27 (22%) of the mock-vaccinated crocodiles developed skin lesions characteristic of WNV infection (Fig. 2C). Four of these animals (14.8%) had one or more lesions positive for viral RNA by qRT-PCR. The average viral RNA load per 'pix' lesion by qRT-PCR was 10^4^ TCID_50_ equivalent (Fig. 2D).

### BinJV/WNV_KUN_ vaccination prevents viral shedding into water

Our previous research demonstrated that infected saltwater crocodiles shed the virus into water resulting in fecal-oral transmission of WNV to tankmate crocodiles^7^. Water samples, as well as cloacal swabs from unvaccinated pen mates (*n* = 5 per group), were collected and tested by qRT-PCR to investigate if the BinJV/WNV_KUN_ vaccine prevented virus shedding by vaccinated crocodiles challenged with WNV_KUN_. None of the samples collected from vaccinated animals or their unvaccinated pen mates yielded detectable viral genomic signals by qRT-PCR. In contrast, four cloacal swab samples out of 27 (14.8%) collected from the mock vaccinated group 14 days post-challenge were positive by qRT-PCR. Two of the four shedding animals also had 'pix' skin lesions. The water samples collected from the mock vaccinated group on days 10 and 13 post-challenge were also positive for WNV_KUN_ RNA by qRT-PCR (Supplementary Tables 5, 6). In contrast, water samples from all vaccinated groups were negative at all sampling time points (Supplementary Table 6).

### Mosquito surveillance throughout the vaccine trials

All FTA cards were negative for WNV_KUN_ by qRT-PCR, and no mosquitoes were caught within or around the experimental facilities during the experiments. These findings confirm that there were few mosquitoes in the environment and that no natural mosquito-borne viral transmission was detected during the studies.

## Discussion

West Nile virus is an economic threat to the crocodile farming industry due to the skin lesions (pix) caused by the virus. Although there are WNV vaccines for veterinary use in equines, and one is used off-label in alligators in the USA, there are no published reports of the vaccine efficacy in American alligators. Currently, there is no vaccine available for crocodilians in Australia, South-East Asia, and Africa, where the risk of new WNV outbreaks remains imminent and significant. Here, we demonstrate that the BinJV/WNV_KUN_ vaccine induces a robust, humoral immune response after two vaccinations administered subcutaneously or intramuscularly at 4 weeks interval. We also demonstrate that the BinJV/WNV_KUN_ vaccine offers complete protection from infection and skin lesions in challenged saltwater crocodiles. This is the first report of a vaccine that protects reptiles against viral infection.

The efficacy of BinJV-based chimeric vaccines in the prevention of flavivirus infections has previously been demonstrated in various mouse models^21–24^. Here we demonstrate for the first time in a non-murine species the efficacy and safety of BinJV/WNV_KUN_ as a vaccine to prevent infection and WNV-induced skin lesions in farmed saltwater crocodiles.

The immune response in reptiles, i.e. cold-blooded animals, is generally slower to develop than in mammalian and avian species^25^. This may explain the late increase in antibody titre, beyond 8 weeks post booster immunisation with BinJV/WNV_KUN_ in the on-farm vaccine immunogenicity study. Another alternative explanation, that a boost to the immune response prior to the 6-months blood sampling may have occurred via natural exposure to WNV_KUN_, is unlikely since no mosquito activity or WNV transmission was detected around the experimental area during the course of the trial—conducted during the tropical dry season. Furthermore, there was no evidence of natural infection as the animals remained negative in the WNV_KUN_ NS1 blocking-ELISA (Supplementary Table 7) and mock-vaccinated crocodiles had no neutralising antibodies at these time points. Hence, a more gradual, immune maturation is the most likely cause for the lengthier titre rise in these animals.

The route of administration and dose of BinJV/WNV_KUN_ did not appear to result in a statistically significant difference in the neutralising antibody response within the dose range tested. This provides more options to the vaccinator for selecting the dosage and the most convenient route of administration of the vaccine under a particular set of farm management conditions.

While the live vaccine induced slightly higher median VNT titres than the inactivated form and seroconversion was detected in more animals, the differences were not statistically significant (Supplementary Fig. 4). The addition of Advax^TM^ adjuvant to both the live and inactivated vaccine formulations increased the median VNT titres, however, again the differences were not statistically significant (Fig. 2B). Advax™ is a GMP-grade delta-inulin polysaccharide-based adjuvant that has been proven to be highly potent^26,27^. It has been demonstrated that Advax^TM^ enhances the cellular immune response, mainly T-cell responses and B-cell memory, when co-administered with an immunogen of interest^28–31^. This study did not assess the cellular immune response following vaccination with BinJV/WNV_KUN_; therefore, it would be interesting to investigate that component of the immune response, particularly looking at the potential mid- to long-term protection associated with immunological memory in both humoral and cellular immune responses^32^. Nevertheless, we observed full protection against 'pix' skin lesions irrespective of whether the vaccine was inactivated or live or formulated with adjuvant or not when animals were challenged with the virus 4 weeks post booster vaccination. These results are consistent with findings from our previous studies with this vaccine in mice^22^.

No residual BinJV/WNV_KUN_ vaccine was detected in the blood of vaccinated animals 2 weeks post-vaccination. This is in contrast to reports that a commercially available inactivated equine vaccine (West Nile Virus Vaccine, Boehringer Ingelheim Vetmedica, Inc. [BIVI], St. Joseph, MO) used off-label in American alligators, remained detectable for weeks after the vaccination raising some safety concerns^20^.

Since, for ethical reasons, we were only able to bleed the animals every three days in the vaccination-challenge study, we may, by chance, have missed detecting viraemia in some crocodiles in the unvaccinated control group at 2 days post-challenge. Alternatively, the virus titres might have been below the limit of detection at that time point. Similarly, the 40% of animals with detectable viraemia at 4 days post-challenge may only represent the minimum frequency of viraemia in unvaccinated animals. We have previously demonstrated that viraemia is brief in most crocodiles^7^; therefore, a snapshot sampling does not necessarily represent the full extent of viraemia in infected animals. Notably, some apparently non-viraemic crocodiles from the control group developed typical pix skin lesions, supporting the contention of more widespread systemic infection than detected in the selective blood sampling and testing.

Only 14.8% of the cloacal swabs collected from the unvaccinated control group 14 days post-challenge tested positive for WNV by qRT-PCR. This proportion is also likely to be lower than the actual number of crocodiles shedding the virus into water. We previously demonstrated that infected animals start shedding the virus between days 3- and 21 post-infection and shedding can be intermittent in a single host^7^. Therefore, we hypothesise that earlier sampling could have resulted in a higher proportion of positive swabs in unvaccinated animals.

Overall, the undetectable levels of the virus in the blood or cloacal swabs of vaccinated crocodiles after the WNV_KUN_ challenge, the absence of the virus in the pen water of these animals and the lack of transmission to their unvaccinated pen mates, suggests that sterilising immunity was achieved through vaccination, with no virus replication occurring after challenge. Nevertheless, we do acknowledge that low levels of virus replication, below the level of sensitivity of our assays, may have occurred in the vaccinated animals. This is suggested by a rise in neutralising antibody levels after the virus challenge, although this could also be due to a response to the 'input antigen' in the challenge dose and/or a delay in the peak antibody response to vaccination, as observed in the initial experiment.

This is the first study to demonstrate the efficacy and safety of the BinJV/WNV_KUN_ chimeric vaccine candidate in a species other than mice. The study also provides the first documented evidence of a vaccine that protects crocodilians against viraemia, fecal virus shedding and 'pix' skin lesions caused by WNV infection. In addition to its efficacy, BinJV/WNV_KUN_ was also confirmed not to replicate to detectable levels in vaccinated crocodiles, consistent with our previous published reports that these chimeric vaccines do not replicate in vertebrate cells^21,33^. Therefore, BinJV/WNV_KUN_ provides an excellent vaccine platform for use in farmed crocodiles as it does not pose public health or environmental risks^7,34^. The vaccine also represents a promising WNV vaccine for other farmed crocodilians, including American alligators and Nile crocodiles (*Crocodylus niloticus*).

Based on the data reported here and elsewhere^22–24^, and additional unpublished studies, the BinJV/WNV_KUN_ vaccine and other vaccines based on this platform, are currently being assessed by Australian regulatory authorities for safety and efficacy prior to approval of the vaccine platform for commercial manufacture for veterinary applications.

## Methods

### Ethical consideration

This study was conducted in accordance with the University of Queensland research ethical guidelines. Ethical approval for the studies was obtained from the University of Queensland’s Native and Exotic Wildlife and Marine Animal Ethics Committee (SCMB/028/17, SCMB/551/18, SCMB/515/19). During vaccination and sample collection, animal care and use protocols adhered to the Animal Welfare Regulations 2000 of the Northern Territory of Australia, *The Code of Practise on the Humane Treatment of Wild and Farmed Australian Crocodiles*, and the *Australian Code for the Care and Use of Animals for Scientific Purposes*. Further, the Centre for Crocodile Research operates under research licence 061 from the Northern Territory Government’s Department of Industry, Tourism and Trade and the Office of the Gene Technology Regulator (OGTR), Australia (Approval # DIR-159).

### Chimeric viruses as vaccine candidates

Chimeric vaccine candidates comprising the genome backbone of the insect-specific BinJV and genes for the structural pre-membrane and envelope (prM-E) proteins of the Kunjin strain of West Nile virus (WNV_KUN_) were generated by circular polymerase extension reaction (CPER). The chimera replication competency was confirmed by immunofluorescence tests (IFA) of transfected C6/36 cells^21^. These chimeric viruses either contained the sequence for prM-E from a 2011 isolate^35^ (designated BinJV/WNV_KUN2011_) or contained the prM-E sequence from the 1960 prototype isolate^36^ (designated as BinJV/WNV_KUNproto_). These two viruses were assessed for safety and immunogenicity in crocodiles. Vaccine doses, routes of administration, number of immunisations and number of vaccinated animals per treatment group are summarised in Tables 2, 3.

**Table 3:** Vaccine formulations used in the challenge study.

Treatment group/Vaccine	Type	Dose	Route	Number of immunisations	Number of vaccinated animals	Unvaccinated pen mates	
BinJV/WNV_KUN2011_	Chimeric vaccine	10 µg live	IM	2	25	5	
		10 µg inactivated	IM	2	25	5	
		10 µg live adjuvanted	IM	2	25	5	
		10 µg inactivated adjuvanted	IM	2	25	5	
Mock vaccinated	PBS	-	IM	2	27	-	

### Animals and experimental design

Saltwater crocodile hatchlings, from different clutches, were screened for maternal antibodies against WNV at four months of age both in blocking ELISA using pan-flavivirus E protein reactive monoclonal antibody 6B6C-1 complemented by WNV NS1 specific reactive monoclonal antibody 3.1112G and in virus neutralisation test (VNT) in Vero cells^7,37^. Hatchlings free from maternal antibodies against WNV were housed in BSL2 and OGTR-approved facilities in a mosquito-free environment with water and air temperature thermostatically controlled by heaters (32 ^o^C ± 1–2). Other husbandry practices, including feeding and hygiene, were also adhered to^7^. The crocodile hatchlings were individually identified and randomly assigned to different treatment groups based on the clutch of origin, vaccine type and regimen. The number of animals per group was determined based on a Statistical Power of 0.8 and α of 0.05 calculated using PS: Power and Sample Size Calculation version 3.1 software. We first assessed the immunogenicity and safety of BinJV/WNV_KUNproto_ and BinJV/WNV_KUN2011_ (Table 1) and then investigated the efficacy of BinJV/WNV_KUN2011_ in a challenge study (Table 3).

### Vaccine preparation

For vaccine preparation, confluent C6/36 cell monolayers, grown in stationary T175 culture flasks (Greiner BioOne GmbH, Germany) in RPMI medium containing 2% fetal bovine serum, were infected with the virus at a multiplicity of infection (MOI) of 0.1 and incubated at 28 ^o^C. The virus supernatant was collected at 5 dpi, clarified by centrifuging at 1680×*g*, 4 ^o^C for 30 min, and stored at 4 ^o^C until purification. The infected cell monolayers were replenished with fresh medium, and the harvesting process was repeated every 48–72 h for a maximum of five harvests.

### Vaccine purification

Polyethylene glycol 8000 (PEG 8000; Sigma-Aldrich, USA) w/v in NTE buffer [10 mM Tris, 1 mM EDTA and 120 mM NaCl; pH 8.0]) was added to the virus supernatant 1:4 (v:v) and stirred slowly overnight at 4 ^o^C. The mixture was then centrifuged at 11,900×*g* for 90 min at 4 ^o^C (Beckman-Coulter JLA10.500 rotor) to pellet the virus. Following this, a sucrose cushion (20% sucrose in NTE, w/v) was layered under the resuspended pellet and the preparation was centrifuged at 133,668×*g* (Beckman-Coulter, SW32Ti rotor) for 2 h at 4 ^o^C. The sucrose and supernatant were discarded, and the virus precipitate was soaked in NTE overnight at 4 ^o^C. The following day, the pellet was carefully resuspended and layered onto a 25–40% potassium tartrate gradient in open-top thin-walled ultra-clear centrifuge tubes (11 × 60 mm, Beckman-Coulter) and centrifuged at 336,238×*g* (Beckman-Coulter, SW60Ti rotor) for 1 h at 4 ^o^C. The virus bands were collected, and the purified virus buffer was exchanged into sterile PBS using 30 kDa Amicon Ultra-15 centrifugal filter units (Merck), followed by storage at 4 ^o^C until required. Purified virions were quantified with respect to envelope protein content against BSA standards by SDS-PAGE, Sypro Ruby staining (Invitrogen™) and ImageJ analysis^22^.

### Vaccine inactivation

Vaccine inactivation was achieved by exposing the vaccine to ultraviolet-C (UV-C) radiation for the optimal time^22^. Briefly, vaccine stock was diluted in sterile 1x PBS (pH 7.4) to the working stock (10 µg in 100 µL). In a 24-well plate, 250 µL of the vaccine was aliquoted into each well, and the plates were placed on ice in a biosafety cabinet. With the lid off the plate, the vaccine was exposed to UV-C light for 90 min. The inactivated vaccine was titrated by the TCID_50_ method on C6/36 cells to ensure the efficacy of UV-C inactivation. The vaccine inactivation was confirmed when the titre was below the limit of detection by TCID_50_ assay (2.30 log_10_ TCID_50_/ml), indicating the vaccine was at least 99.99% inactivated^22^. Both live and inactivated vaccines were stored at 4 ^o^C until they were used.

### Immunogenicity and safety study with BinJV/WNV_KUN_ chimeric vaccine candidates

Four-month-old crocodile hatchlings, free of anti-WNV maternal antibodies, were immunised with each vaccine candidate either subcutaneously (SC) or intramuscularly (IM) (Table 1) in order to test their immunogenicity and safety. A subset (20% of animals in each group) of vaccinated and mock vaccinated animals were blood sampled at time points indicated in Fig. 1A, and tested for the residual BinJV/WNV vaccine as per OGTR requirements. Blood plasma was heat-inactivated and tested in VNT for seroconversion to WNV_KUN2011_ antigens^22^. A virus micro-neutralisation test (VNT) was performed in a 96-well plate using Vero cells. Heat-inactivated sera were titrated twofold in plain DMEM cell culture media. WNV_KUN2011_ was diluted in DMEM supplemented with 2% FBS, one-time penicillin–streptomycin, and 2 mM l-glutamine. Hundred infectious units of diluted WNV_KUN_ were added to the titrated serum samples. The mixture was incubated for 1 h in 37 ^o^C incubator with 5% CO_2_. The virus neutralisation titre was determined as a reciprocal value of the highest serum dilution in which there was no virus replication. The lowest sample dilution was 1 in 20, while the highest dilution was 1 in 2560. The lack of virus replication was confirmed in a fixed cell ELISA using the pan-flavivirus E reactive monoclonal antibody 4G2. Animals were monitored daily for any potential vaccine-related adverse effects. Pen water samples were collected and tested for the presence of the BinJV/WNV_KUN_ viral genome.

### Vaccination with BinJV/WNV_KUN_ and WNV_KUN_ challenge study

After the immunogenicity and safety study, a challenge study was conducted where 4 months old crocodile hatchlings, determined to be free of anti-WNV maternal antibodies were allocated to five treatment groups, each group housed in a separate pen. Two groups were vaccinated with 10 µg of purified live BinJV/WNV_KUN_ with or without 1 mg Advax™ adjuvant (Vaxine Pty Ltd., Adelaide, SA), while two other groups were immunised with 10 µg purified UV-inactivated BinJV/WNV_KUN_ with or without 1 mg Advax™ adjuvant. The control group received a placebo (PBS). All vaccine formulations were administered twice, four weeks apart, intramuscularly in a total volume of 120 µL (Table 3). Twelve weeks post initial vaccination (4 weeks post booster vaccination), all animals were subcutaneously challenged with 1 × 10^5^ infectious units of WNV_Kun_ (NSW 2011 strain JN887352) as described^7^.

Blood and cloacal swab samples were collected 14 days post booster vaccination to test for the residual vaccine in vaccinated crocodiles or evidence of chimeric vaccine replication. Water samples were also collected daily from each treatment tank to assess for potential vaccine shedding into water. Five animals in each pen of the vaccine groups were left unvaccinated and unchallenged. These animals served as tankmate controls to determine if vaccinated animals would still shed the challenge virus into the pen water and transmit it to unvaccinated crocodiles via the fecal-oral route as previously demonstrated for unvaccinated animals^7^. Following vaccination and challenge, animals were monitored daily for occurrence of adverse reactions post-vaccination, or development of clinical signs following the virus challenge. Experimental animals were blood sampled on the day of vaccination (day zero, baseline sampling), 4 weeks (i.e., at the time of booster vaccination), 8 weeks (4 weeks post booster vaccination and time of virus challenge), and finally 13 weeks post initial vaccination and 5 weeks post-challenge (Fig. 2A).

At 2- and 4-days post-challenge, blood samples were collected from 10 (40%) randomly selected animals from each treatment group. These samples were tested for viral RNA in the blood (TCID_50_/ml equivalents) by qRT-PCR. Cloacal swab samples were collected at days 0 and 14 post-challenge and similarly tested by qRT-PCR. Five weeks post-challenge, animals from all treatment groups, including the mock vaccinated group were blood sampled and examined for the presence of 'pix' skin lesions. Mock vaccinated were euthanised and cloacal swab samples were collected. After euthanasia, a systematic post-mortem examination was performed, including collecting skin samples with and without 'pix' lesions for viral genome investigation by WNV_KUN_ qRT-PCR. Crocodile skin was examined according to the existing protocols for the presence of 'pix' lesions^15^.

CO_2_-baited mosquito traps (SMACK traps)^38^ equipped with honey-baited nucleic acid preservation cards (FTA^TM^ cards) were installed around the experimental area to monitor for the baseline of natural mosquito transmission of WNV_KUN_ and other arboviruses for the duration of the trial^39,40^. The FTA card surveillance was conducted within the PC2 facility for the vaccine challenge study. For the on-farm vaccinations, the traps were set near the crocodile pens and other strategic sites on the farm.

### RNA extraction

Viral RNA was extracted from plasma and swab samples using the Machery–Nagel Viral RNA Isolation kit (Dueren, Germany) as per the manufacturer’s instructions. Total RNA from skin tissue samples was isolated using the RNeasy Plus Kit (Qiagen, Inc.). Viral RNA from the water was isolated using RNeasy PowerWater Kit (Qiagen, Inc.) in accordance with manufacturer instructions. RNA was extracted from FTA cards using TRIzol Reagent (Life Technologies) according to the manufacturer’s guidelines with a few modifications by ref. ^41^.

### WNV RNA quantitation (qRT-PCR) in challenged animals

Viral genome load quantitation was achieved by quantitative reverse-transcriptase PCR (qRT-PCR) using QIAGEN’s real-time PCR cycler, the Rotor-Gene Q (Qiagen, Inc.). Invitrogen™ SuperScript™ III Platinum™ Taq One-Step qRT-PCR System Kit (Life Technologies Corporation, Carlsbad, USA) was used. The WNV genome quantitation in plasma samples was determined using a WNV_KUN_ RNA standard derived from a viral stock with known titre^7^. For cloacal swabs and water, the assessment was dichotomic based on the presence (positive) or absence (negative) of viral RNA in tested samples.

The qRT-PCR reaction consisted of 15 µL of the optimised master mix and 5 µL of RNA template (Supplementary Table 1). The cycling conditions consisted of cDNA synthesis at 55 °C for 5 min, PCR initial activation at 95 ^o^C for 2 min, followed by 50 cycles of 95 ^o^C for 3 s, and 60 ^o^C for 30 s. For each run, three technical replicates for each sample, a positive control, a negative control, and a no-template control were used. A positive sample was determined based on the CT value corresponding to the assay’s limit of detection^7^. A sample was considered negative based on the absence of a qRT-PCR amplification signal or a CT value greater than the limit of detection (Supplementary Fig. 2).

### Investigation of the presence of vaccine residues in plasma samples from vaccinated animals (one-step RT-PCR and two-step qRT-PCR)

A one-step RT-PCR was used to test for BinJV/WNV_KUN_ vaccine in plasma samples from vaccinated animals, water, and cloacal swabs using Invitrogen™ SuperScript™ III Platinum™ Taq One-Step qRT-PCR System Kit (Life Technologies Corporation, Carlsbad, USA) and primers targeting BinJV NS5 (forward primer 5′-GCAAGATGTACGCCGATGACACCGC-3′ and a reverse primer 5′-GCCATGTCGTTTAGATAGGTGAGAGC-3′) amplifying a 480 base pairs sequence. The RT-PCR reaction consisted of 10 µL of the master mix and 2.5 µL of the RNA template (Supplementary Table 2). The PCR cycling conditions were one cycle at 45 °C for 30 min, one cycle at 94 °C for 2 min, 40 cycles at 94 °C for 30 s, 45 °C for 30 s, and 68 °C for 1 min and one cycle at 68 °C for 10 min. PCR products were run on 2% agarose gel electrophoresis. The size of the amplicon was verified using Bioline HyperLadder™ 1 kb (Meridian Bioscience, Australia).

The same samples were also tested with the two-step qRT-PCR using QuantiNova SYBR Green PCR Kit (Qiagen, Inc.) according to the manufacturer’s instructions (Supplementary Table 3). The first step consisted of making cDNA using qScript cDNA Synthesis Kit (Quantabio, QIAGEN Beverly, Inc., USA). The cDNA synthesis consisted of a 20 µL reaction containing 4 µL of 5x qScript reaction mix (containing a mix of oligo-dT and random primers), 1 µL 20x qScript reverse transcriptase, 4 µL of RNA template, 1 µL RNAseOUT® (Thermo Fisher, USA) and 10 µL of Nuclease-free water. The RT cycling conditions were one cycle at 22 °C for 5 min, one cycle at 42 °C for 30 min, and one cycle at 85 °C for 5 min. The second step consisted of qPCR using forward primer 5′-ACTGACAGAACTTGGTGCTATG-3′ and reverse primer 5′-GCATACGCCTCTCTCCATTAAG-3′ targeting BinJV NS3. These primers yielded an amplicon of 103 base pairs. The cycling conditions were one cycle at 95 °C for 2 min followed by 40 cycles at 95 °C for 5 s and 60 °C for 10 s. The melting curve analysis was done between 72 and 95 °C. A standard of known BinJV/WNV_KUN_ vaccine titre (by TCID_50_) was assessed simultaneously as a standard in each qPCR assay. Positive, negative, and no-template controls were used for each qPCR run.

### Statistical analysis

Data analysis and generation of graphs were done using GraphPad Prism 9 (GraphPad Software, Inc., San Diego, CA, USA) for Windows (version 9.5.0 (730) 2022). Levels of neutralising antibodies in different treatment groups were statistically analysed using at two-way analysis of variance (ANOVA). The two-way ANOVA was performed for multiple comparison analysis with the α-level set at 0.05 with a Tukey’s post-test.

### Reporting summary

Further information on research design is available in the Nature Research Reporting Summary linked to this article.

## Supplementary information

REPORTING SUMMARY

Supplementary Info
