# Supplementary material for: A chimeric vaccine protects farmed saltwater crocodiles from West Nile virus-induced skin lesions
Source: NPJ Vaccines. 2023 Jun 27;8:93. doi: 10.1038/s41541-023-00688-w (PMC10300036; doi:10.1038/s41541-023-00688-w)
Supplement: Supplementary file 2 — Supplementary Info [file 41541_2023_688_MOESM2_ESM.docx]

**SUPPLEMENTARY TABLES**

Supplementary Table 1. qRT-PCR reaction mix

| **Reagent** | **Volume/Rx. (µL)** |
| --- | --- |
| Nuclease-free water | 2.16 |
| 2x Reaction Mix | 10 |
| Forward primer, 10 μM (AACCCCAGTGGAGAAGTGGA) | 0.4 |
| Reverse primer, 10 μM (TCAGGCTGCCACACCAAA) | 0.8 |
| Fluorogenic probe, 10 μM (6FAM – CGATGTTCCATACTCTGG – MGB NFQ) | 0.2 |
| ROX™ Reference Dye | 0.04 |
| RNaseOUT™ Ribonuclease Inhibitor | 1 |
| SuperScript™ III RT/Platinum™ Taq Mix | 0.4 |
| RNA Template (total RNA) | 5 |
| **Final volume** | **20** |

Supplementary Table 2. BinJV RT-PCR reaction mix

| **Reagent** | **Volume/Rx. (µL)** |
| --- | --- |
| 2x reaction buffer | 6.25 |
| Forward primer (5uM) | 0.5 |
| Reverse primer (5uM) | 0.5 |
| Reverse transcriptase Platinum Taq polymerase | 0.5 |
| Nuclease-free water | 2.25 |
| RNA | 2.5 |
| **Final volume** | **12.5** |

Supplementary Table 3. BinJV qPCR reaction mix

| **Reagent** | **Volume/ Rx. (µL)** | **Final concentration** |
| --- | --- | --- |
| NFH_2_O | 5.2 | - |
| 2x QuantiNova SYBR Green PCR Master Mix | 10 | 1x |
| Forward Primer | 1.4 | 0.7 µM |
| Reverse Primer | 1.4 | 0.7 µM |
| Template (cDNA) | 2 | ≤ 100 ng |
| **Total** | **20** |  |

**Supplementary Table 4. WNV_KUN_ qRT-PCR on randomly collected post-challenge blood samples**

| **No.** | **Samples ID** | **Treatment group** | **CT value** | **Verdict** |
| --- | --- | --- | --- | --- |
| 1 | 4P-5-01 | Mock vaccinated | NS | Negative |
| 2 | 4P-5-01 | Mock vaccinated | NS | Negative |
| 3 | 4P-5-01 | Mock vaccinated | NS | Negative |
| 4 | 4P-5-02 | Mock vaccinated | NS | Negative |
| 5 | 4P-5-02 | Mock vaccinated | NS | Negative |
| 6 | 4P-5-02 | Mock vaccinated | NS | Negative |
| 7 | 4P-5-03 | Mock vaccinated | 37.26 | Positive |
| 8 | 4P-5-03 | Mock vaccinated | 38.55 | Positive |
| 9 | 4P-5-03 | Mock vaccinated | 39.09 | Positive |
| 10 | 4P-5-04 | Mock vaccinated | NS | Negative |
| 11 | 4P-5-04 | Mock vaccinated | NS | Negative |
| 12 | 4P-5-04 | Mock vaccinated | NS | Negative |
| 13 | 4P-5-05 | Mock vaccinated | NS | Negative |
| 14 | 4P-5-05 | Mock vaccinated | NS | Negative |
| 15 | 4P-5-05 | Mock vaccinated | NS | Negative |
| 16 | 4P-5-06 | Mock vaccinated | NS | Negative |
| 17 | 4P-5-06 | Mock vaccinated | NS | Negative |
| 18 | 4P-5-06 | Mock vaccinated | NS | Negative |
| 19 | 4P-5-07 | Mock vaccinated | NS | Negative |
| 20 | 4P-5-07 | Mock vaccinated | 46.2 | +/- |
| 21 | 4P-5-07 | Mock vaccinated | NS | Negative |
| 22 | 4P-5-08 | Mock vaccinated | 32.68 | Positive |
| 23 | 4P-5-08 | Mock vaccinated | 32.02 | Positive |
| 24 | 4P-5-08 | Mock vaccinated | 32.6 | Positive |
| 25 | 4P-5-09 | Mock vaccinated | 29.64 | Positive |
| 26 | 4P-5-09 | Mock vaccinated | 29.58 | Positive |
| 27 | 4P-5-09 | Mock vaccinated | 29.57 | Positive |
| 28 | 4P-5-10 | Mock vaccinated | 30.69 | Positive |
| 29 | 4P-5-10 | Mock vaccinated | 30.26 | Positive |
| 30 | 4P-5-10 | Mock vaccinated | 31.58 | Positive |
| 31 | 4P-4-01 | Inactivated + Adjuvant | NS | Negative |
| 32 | 4P-4-01 | Inactivated + Adjuvant | NS | Negative |
| 33 | 4P-4-01 | Inactivated + Adjuvant | NS | Negative |
| 34 | 4P-4-02 | Inactivated + Adjuvant | NS | Negative |
| 35 | 4P-4-02 | Inactivated + Adjuvant | NS | Negative |
| 36 | 4P-4-02 | Inactivated + Adjuvant | NS | Negative |
| 37 | 4P-4-03 | Inactivated + Adjuvant | NS | Negative |
| 38 | 4P-4-03 | Inactivated + Adjuvant | NS | Negative |
| 39 | 4P-4-03 | Inactivated + Adjuvant | NS | Negative |
| 40 | 4P-4-04 | Inactivated + Adjuvant | NS | Negative |
| 41 | 4P-4-04 | Inactivated + Adjuvant | NS | Negative |
| 42 | 4P-4-04 | Inactivated + Adjuvant | NS | Negative |
| 43 | 4P-4-05 | Inactivated + Adjuvant | NS | Negative |
| 44 | 4P-4-05 | Inactivated + Adjuvant | NS | Negative |
| 45 | 4P-4-05 | Inactivated + Adjuvant | NS | Negative |
| 46 | 4P-4-06 | Inactivated + Adjuvant | NS | Negative |
| 47 | 4P-4-06 | Inactivated + Adjuvant | NS | Negative |
| 48 | 4P-4-06 | Inactivated + Adjuvant | NS | Negative |
| 49 | 4P-4-07 | Inactivated + Adjuvant | NS | Negative |
| 50 | 4P-4-07 | Inactivated + Adjuvant | NS | Negative |
| 51 | 4P-4-07 | Inactivated + Adjuvant | NS | Negative |
| 52 | 4P-4-08 | Inactivated + Adjuvant | NS | Negative |
| 53 | 4P-4-08 | Inactivated + Adjuvant | NS | Negative |
| 54 | 4P-4-08 | Inactivated + Adjuvant | NS | Negative |
| 55 | 4P-4-09 | Inactivated + Adjuvant | NS | Negative |
| 56 | 4P-4-09 | Inactivated + Adjuvant | NS | Negative |
| 57 | 4P-4-09 | Inactivated + Adjuvant | NS | Negative |
| 58 | 4P-4-10 | Inactivated + Adjuvant | NS | Negative |
| 59 | 4P-4-10 | Inactivated + Adjuvant | NS | Negative |
| 60 | 4P-4-10 | Inactivated + Adjuvant | NS | Negative |
| 61 | 4P-3-01 | Inactivated | NS | Negative |
| 62 | 4P-3-01 | Inactivated | NS | Negative |
| 63 | 4P-3-01 | Inactivated | NS | Negative |
| 64 | 4P-3-02 | Inactivated | NS | Negative |
| 65 | 4P-3-02 | Inactivated | NS | Negative |
| 66 | 4P-3-02 | Inactivated | NS | Negative |
| 67 | 4P-3-03 | Inactivated | NS | Negative |
| 68 | 4P-3-03 | Inactivated | NS | Negative |
| 69 | 4P-3-03 | Inactivated | NS | Negative |
| 70 | 4P-3-04 | Inactivated | NS | Negative |
| 71 | 4P-3-04 | Inactivated | NS | Negative |
| 72 | 4P-3-04 | Inactivated | NS | Negative |
| 73 | 4P-3-05 | Inactivated | NS | Negative |
| 74 | 4P-3-05 | Inactivated | NS | Negative |
| 75 | 4P-3-05 | Inactivated | NS | Negative |
| 76 | 4P-3-06 | Inactivated | NS | Negative |
| 77 | 4P-3-06 | Inactivated | NS | Negative |
| 78 | 4P-3-06 | Inactivated | NS | Negative |
| 79 | 4P-3-07 | Inactivated | NS | Negative |
| 80 | 4P-3-07 | Inactivated | NS | Negative |
| 81 | 4P-3-07 | Inactivated | NS | Negative |
| 82 | 4P-3-08 | Inactivated | NS | Negative |
| 83 | 4P-3-08 | Inactivated | NS | Negative |
| 84 | 4P-3-08 | Inactivated | NS | Negative |
| 85 | 4P-3-09 | Inactivated | NS | Negative |
| 86 | 4P-3-09 | Inactivated | NS | Negative |
| 87 | 4P-3-09 | Inactivated | NS | Negative |
| 88 | 4P-3-10 | Inactivated | NS | Negative |
| 89 | 4P-3-10 | Inactivated | NS | Negative |
| 90 | 4P-3-10 | Inactivated | NS | Negative |
| 91 | 4P-2-01 | Live + Adjuvant | NS | Negative |
| 92 | 4P-2-01 | Live + Adjuvant | NS | Negative |
| 93 | 4P-2-01 | Live + Adjuvant | NS | Negative |
| 94 | 4P-2-02 | Live + Adjuvant | NS | Negative |
| 95 | 4P-2-02 | Live + Adjuvant | NS | Negative |
| 96 | 4P-2-02 | Live + Adjuvant | NS | Negative |
| 97 | 4P-2-03 | Live + Adjuvant | NS | Negative |
| 98 | 4P-2-03 | Live + Adjuvant | NS | Negative |
| 99 | 4P-2-03 | Live + Adjuvant | NS | Negative |
| 100 | 4P-2-04 | Live + Adjuvant | NS | Negative |
| 101 | 4P-2-04 | Live + Adjuvant | NS | Negative |
| 102 | 4P-2-04 | Live + Adjuvant | NS | Negative |
| 103 | 4P-2-05 | Live + Adjuvant | NS | Negative |
| 104 | 4P-2-05 | Live + Adjuvant | NS | Negative |
| 105 | 4P-2-05 | Live + Adjuvant | NS | Negative |
| 106 | 4P-2-06 | Live + Adjuvant | NS | Negative |
| 107 | 4P-2-06 | Live + Adjuvant | NS | Negative |
| 108 | 4P-2-06 | Live + Adjuvant | NS | Negative |
| 109 | 4P-2-07 | Live + Adjuvant | NS | Negative |
| 110 | 4P-2-07 | Live + Adjuvant | NS | Negative |
| 111 | 4P-2-07 | Live + Adjuvant | NS | Negative |
| 112 | 4P-2-08 | Live + Adjuvant | NS | Negative |
| 113 | 4P-2-08 | Live + Adjuvant | NS | Negative |
| 114 | 4P-2-08 | Live + Adjuvant | NS | Negative |
| 115 | 4P-2-09 | Live + Adjuvant | NS | Negative |
| 116 | 4P-2-09 | Live + Adjuvant | NS | Negative |
| 117 | 4P-2-09 | Live + Adjuvant | NS | Negative |
| 118 | 4P-2-10 | Live + Adjuvant | NS | Negative |
| 119 | 4P-2-10 | Live + Adjuvant | NS | Negative |
| 120 | 4P-2-10 | Live + Adjuvant | NS | Negative |
| 121 | 4P-1-01 | Live | NS | Negative |
| 122 | 4P-1-01 | Live | NS | Negative |
| 123 | 4P-1-01 | Live | NS | Negative |
| 124 | 4P-1-02 | Live | NS | Negative |
| 125 | 4P-1-02 | Live | NS | Negative |
| 126 | 4P-1-02 | Live | NS | Negative |
| 127 | 4P-1-03 | Live | NS | Negative |
| 128 | 4P-1-03 | Live | NS | Negative |
| 129 | 4P-1-03 | Live | NS | Negative |
| 130 | 4P-1-04 | Live | NS | Negative |
| 131 | 4P-1-04 | Live | NS | Negative |
| 132 | 4P-1-04 | Live | NS | Negative |
| 133 | 4P-1-05 | Live | NS | Negative |
| 134 | 4P-1-05 | Live | NS | Negative |
| 135 | 4P-1-05 | Live | NS | Negative |
| 136 | 4P-1-06 | Live | NS | Negative |
| 137 | 4P-1-06 | Live | NS | Negative |
| 138 | 4P-1-06 | Live | NS | Negative |
| 139 | 4P-1-07 | Live | NS | Negative |
| 140 | 4P-1-07 | Live | NS | Negative |
| 141 | 4P-1-07 | Live | NS | Negative |
| 142 | 4P-1-08 | Live | NS | Negative |
| 143 | 4P-1-08 | Live | NS | Negative |
| 144 | 4P-1-08 | Live | NS | Negative |
| 145 | 4P-1-09 | Live | NS | Negative |
| 146 | 4P-1-09 | Live | NS | Negative |
| 147 | 4P-1-09 | Live | NS | Negative |
| 148 | 4P-1-10 | Live | NS | Negative |
| 149 | 4P-1-10 | Live | NS | Negative |
| 150 | 4P-1-10 | Live | NS | Negative |
| 200 | KUNV 10^5.8 | Standard | 19.05 | Positive |
| 201 | KUNV 10^4.8 | Standard | 22.71 | Positive |
| 202 | KUNV 10^3.8 | Standard | 26.53 | Positive |
| 203 | KUNV 10^2.8 | Standard | 29.8 | Positive |
| 204 | BinJV/NHUV | Negative Control | NS | Negative |
| 205 | NFH_2_O | NTC | NS | Negative |

**Supplementary Table 5. WNV_KUN_ qRT-PCR on cloacal swabs and pen water samples**

| **No.** | **Samples ID** | **Treatment group** | **Sample type** | **Ct value** | **Verdict** |
| --- | --- | --- | --- | --- | --- |
| 1 | CS2-F4 | Mock vaccinated | Cloacal swabs | NS | Negative |
| 2 | CS2-F4 | Mock vaccinated | Cloacal swabs | NS | Negative |
| 3 | CS2-F4 | Mock vaccinated | Cloacal swabs | NS | Negative |
| 4 | CS2-F16 | Mock vaccinated | Cloacal swabs | 49.51 | +/- |
| 5 | CS2-F16 | Mock vaccinated | Cloacal swabs | NS | Negative |
| 6 | CS2-F16 | Mock vaccinated | Cloacal swabs | NS | Negative |
| 7 | CS2-F33 | Mock vaccinated | Cloacal swabs | 31.24 | Positive |
| 8 | CS2-F33 | Mock vaccinated | Cloacal swabs | 31.45 | Positive |
| 9 | CS2-F33 | Mock vaccinated | Cloacal swabs | 31.10 | Positive |
| 10 | CS2-F44 | Mock vaccinated | Cloacal swabs | NS | Negative |
| 11 | CS2-F44 | Mock vaccinated | Cloacal swabs | NS | Negative |
| 12 | CS2-F44 | Mock vaccinated | Cloacal swabs | NS | Negative |
| 13 | CS2-D12 | Mock vaccinated | Cloacal swabs | 34.65 | Positive |
| 14 | CS2-D12 | Mock vaccinated | Cloacal swabs | 35.54 | Positive |
| 15 | CS2-D12 | Mock vaccinated | Cloacal swabs | 31.83 | Positive |
| 16 | CS2-H19 | Mock vaccinated | Cloacal swabs | NS | Negative |
| 17 | CS2-H19 | Mock vaccinated | Cloacal swabs | NS | Negative |
| 18 | CS2-H19 | Mock vaccinated | Cloacal swabs | NS | Negative |
| 19 | CS2-B44 | Mock vaccinated | Cloacal swabs | 32.02 | Positive |
| 20 | CS2-B44 | Mock vaccinated | Cloacal swabs | 32.88 | Positive |
| 21 | CS2-B44 | Mock vaccinated | Cloacal swabs | 31.42 | Positive |
| 22 | CS2-C32 | Mock vaccinated | Cloacal swabs | NS | Negative |
| 23 | CS2-C32 | Mock vaccinated | Cloacal swabs | NS | Negative |
| 24 | CS2-C32 | Mock vaccinated | Cloacal swabs | NS | Negative |
| 25 | CS2-F1 | Inactivated + Adjuvant | Cloacal swabs | NS | Negative |
| 26 | CS2-F1 | Inactivated + Adjuvant | Cloacal swabs | NS | Negative |
| 27 | CS2-F1 | Inactivated + Adjuvant | Cloacal swabs | NS | Negative |
| 28 | CS2-F8 | Inactivated + Adjuvant | Cloacal swabs | NS | Negative |
| 29 | CS2-F8 | Inactivated + Adjuvant | Cloacal swabs | NS | Negative |
| 30 | CS2-F8 | Inactivated + Adjuvant | Cloacal swabs | NS | Negative |
| 31 | CS2-F25 | Inactivated + Adjuvant | Cloacal swabs | NS | Negative |
| 32 | CS2-F25 | Inactivated + Adjuvant | Cloacal swabs | NS | Negative |
| 33 | CS2-F25 | Inactivated + Adjuvant | Cloacal swabs | NS | Negative |
| 34 | CS2-A10 | Inactivated + Adjuvant | Cloacal swabs | NS | Negative |
| 35 | CS2-A10 | Inactivated + Adjuvant | Cloacal swabs | NS | Negative |
| 36 | CS2-A10 | Inactivated + Adjuvant | Cloacal swabs | NS | Negative |
| 37 | CS2-A14 | Inactivated + Adjuvant | Cloacal swabs | NS | Negative |
| 38 | CS2-A14 | Inactivated + Adjuvant | Cloacal swabs | NS | Negative |
| 39 | CS2-A14 | Inactivated + Adjuvant | Cloacal swabs | NS | Negative |
| 40 | CS2-A20 | Inactivated + Adjuvant | Cloacal swabs | NS | Negative |
| 41 | CS2-A20 | Inactivated + Adjuvant | Cloacal swabs | NS | Negative |
| 42 | CS2-A20 | Inactivated + Adjuvant | Cloacal swabs | NS | Negative |
| 43 | CS2-A30 | Inactivated + Adjuvant | Cloacal swabs | NS | Negative |
| 44 | CS2-A30 | Inactivated + Adjuvant | Cloacal swabs | NS | Negative |
| 45 | CS2-A30 | Inactivated + Adjuvant | Cloacal swabs | NS | Negative |
| 46 | CS2-B15 | Inactivated + Adjuvant | Cloacal swabs | NS | Negative |
| 47 | CS2-B15 | Inactivated + Adjuvant | Cloacal swabs | NS | Negative |
| 48 | CS2-B15 | Inactivated + Adjuvant | Cloacal swabs | NS | Negative |
| 49 | CS2-F5 | Inactivated | Cloacal swabs | NS | Negative |
| 50 | CS2-F5 | Inactivated | Cloacal swabs | NS | Negative |
| 51 | CS2-F5 | Inactivated | Cloacal swabs | NS | Negative |
| 52 | CS2-F29 | Inactivated | Cloacal swabs | NS | Negative |
| 53 | CS2-F29 | Inactivated | Cloacal swabs | NS | Negative |
| 54 | CS2-F29 | Inactivated | Cloacal swabs | NS | Negative |
| 55 | CS2-H11 | Inactivated | Cloacal swabs | NS | Negative |
| 56 | CS2-H11 | Inactivated | Cloacal swabs | NS | Negative |
| 57 | CS2-H11 | Inactivated | Cloacal swabs | NS | Negative |
| 58 | CS2-H15 | Inactivated | Cloacal swabs | NS | Negative |
| 59 | CS2-H15 | Inactivated | Cloacal swabs | NS | Negative |
| 60 | CS2-H15 | Inactivated | Cloacal swabs | NS | Negative |
| 61 | CS2-H17 | Inactivated | Cloacal swabs | NS | Negative |
| 62 | CS2-H17 | Inactivated | Cloacal swabs | NS | Negative |
| 63 | CS2-H17 | Inactivated | Cloacal swabs | NS | Negative |
| 64 | CS2-H36 | Inactivated | Cloacal swabs | NS | Negative |
| 65 | CS2-H36 | Inactivated | Cloacal swabs | NS | Negative |
| 66 | CS2-H36 | Inactivated | Cloacal swabs | NS | Negative |
| 67 | KUNV 10^3.8 | Standard | Virus supernatant | 25.31 | Positive |
| 68 | KUNV 10^2.8 | Standard | Virus supernatant | 30.02 | Positive |
| 69 | KUNV 10^1.8 | Standard | Virus supernatant | 32.95 | Positive |
| 70 | KUNV 10^0.8 | Standard | Virus supernatant | 46.23 | Positive |
| 71 | CASV IC 1 | Negative control | CASV supernatant | NS | Negative |
| 72 | NFH_2_O | NTC | NFH2O | NS | Negative |

**Supplementary Table 6. WNV_KUN_ qRT-PCR on pen water samples and FTA cards**

| **No.** | **Samples ID** | **Treatment group** | **Sample type** | **Type** | **Ct value** | **Verdict** |
| --- | --- | --- | --- | --- | --- | --- |
| 1 | H2O_17-09_5 | Mock vaccinated | Pen water | Unknown | NS | Negative |
| 2 | H2O_20-09_1 | Live | Pen water | Unknown | NS | Negative |
| 3 | H2O_20-09_2 | Live + Adjuvant | Pen water | Unknown | NS | Negative |
| 4 | H2O_20-09_3 | Inactivated | Pen water | Unknown | NS | Negative |
| 5 | H2O_20-09_4 | Inactivated + Adjuvant | Pen water | Unknown | NS | Negative |
| 6 | H2O_20-09_5 | Mock vaccinated | Pen water | Unknown | 46.69 | Negative |
| 7 | H2O_23-09_1 | Live | Pen water | Unknown | NS | Negative |
| 8 | H2O_23-09_2 | Live + Adjuvant | Pen water | Unknown | NS | Negative |
| 9 | H2O_23-09_3 | Inactivated | Pen water | Unknown | NS | Negative |
| 10 | H2O_23-09_4 | Inactivated + Adjuvant | Pen water | Unknown | NS | Negative |
| 11 | H2O_23-09_5 | Mock vaccinated | Pen water | Unknown | 46.69 | Negative |
| 12 | FTA_27-07-21 | NA | Pen water | Unknown | NS | Negative |
| 13 | FTA_12-08-21 | NA | Pen water | Unknown | NS | Negative |
| 14 | FTA_26-08-21 | NA | Pen water | Unknown | NS | Negative |
| 15 | FTA_16-09-21 | NA | Pen water | Unknown | NS | Negative |
| 16 | FTA_23-09-21 | NA | Pen water | Unknown | NS | Negative |
| 17 | FTA_06-10-21 | NA | Pen water | Unknown | NS | Negative |
| 18 | KUNV 10^5.8 | Standard | Virus supernatant | Standard | 25.34 | Positive |
| 19 | KUNV 10^4.8 | Standard | Virus supernatant | Standard | 29.43 | Positive |
| 20 | KUNV 10^3.8 | Standard | Virus supernatant | Standard | 33.40 | Positive |
| 21 | KUNV 10^2.8 | standard | Virus supernatant | Standard | 38.02 | Positive |
| 22 | BinJV/NHUV | Negative Control |  |  | NS | Negative |
| 23 | NFH_2_O | NTC |  |  | NS | Negative |

**Supplementary Table 7. WNV_KUN_ NS1 specific blocking-ELISA**

| **SN** | **Pen #** | **Six months post-booster** | **Treatment group** | **Percentage of inhibition** | **Verdict** |
| --- | --- | --- | --- | --- | --- |
| 1 | 95 | 5-B44 | BinJV/WNV_KUNproto_ 2μg SC | -2.75 | Negative |
| 2 | 95 | 5-B45 | BinJV/WNV_KUNproto_ 2μg SC | 5.48 | Negative |
| 3 | 95 | 5-B46 | BinJV/WNV_KUNproto_ 2μg SC | 15.85 | Negative |
| 4 | 95 | 5-B47 | BinJV/WNV_KUNproto_ 2μg SC | 24.80 | Negative |
| 5 | 95 | 5-E25 | BinJV/WNV_KUNproto_ 2μg SC | 26.72 | Negative |
| 6 | 95 | 5-E45 | BinJV/WNV_KUNproto_ 2μg SC | -7.48 | Negative |
| 7 | 95 | 5-E46 | BinJV/WNV_KUNproto_ 2μg SC | -7.70 | Negative |
| 8 | 95 | 5-E47 | BinJV/WNV_KUNproto_ 2μg SC | 12.61 | Negative |
| 9 | 94 | 5-C01 | BinJV/WNV_KUNproto_ 2μg SC | 11.50 | Negative |
| 10 | 94 | 5-C02 | BinJV/WNV_KUNproto_ 2μg SC | 26.73 | Negative |
| 11 | 94 | 5-C03 | BinJV/WNV_KUNproto_ 2μg SC | -12.18 | Negative |
| 12 | 94 | 5-C04 | BinJV/WNV_KUNproto_ 2μg SC | -8.17 | Negative |
| 13 | 94 | 5-C05 | BinJV/WNV_KUNproto_ 2μg SC | 4.96 | Negative |
| 14 | 94 | 5-C06 | BinJV/WNV_KUNproto_ 2μg SC | 15.24 | Negative |
| 15 | 94 | 5-C07 | BinJV/WNV_KUNproto_ 2μg SC | 26.78 | Negative |
| 16 | 94 | 5-C08 | BinJV/WNV_KUNproto_ 2μg SC | -18.05 | Negative |
| 17 | 94 | 5-C09 | BinJV/WNV_KUNproto_ 2μg SC | -3.25 | Negative |
| 18 | 94 | 5-C10 | BinJV/WNV_KUNproto_ 2μg SC | 8.44 | Negative |
| 19 | 94 | 5-C11 | BinJV/WNV_KUNproto_ 2μg SC | 23.62 | Negative |
| 20 | 94 | 5-C12 | BinJV/WNV_KUNproto_ 2μg SC | 29.07 | Negative |
| 21 | 94 | 5-E26 | BinJV/WNV_KUNproto_ 2μg SC | -3.18 | Negative |
| 22 | 92 | 5-C13 | BinJV/WNV_KUNproto_ 10μg SC | -3.01 | Negative |
| 23 | 92 | 5-C15 | BinJV/WNV_KUNproto_ 10μg SC | 6.34 | Negative |
| 24 | 92 | 5-C17 | BinJV/WNV_KUNproto_ 10μg SC | 17.30 | Negative |
| 25 | 92 | 5-C18 | BinJV/WNV_KUNproto_ 10μg SC | 26.68 | Negative |
| 26 | 92 | 5-C20 | BinJV/WNV_KUNproto_ 10μg SC | -1.48 | Negative |
| 27 | 92 | 5-C24 | BinJV/WNV_KUNproto_ 10μg SC | 5.74 | Negative |
| 28 | 92 | 5-C25 | BinJV/WNV_KUNproto_ 10μg SC | 17.66 | Negative |
| 29 | 92 | 5-C26 | BinJV/WNV_KUNproto_ 10μg SC | -10.50 | Negative |
| 30 | 91 | 5-C27 | BinJV/WNV_KUNproto_ 10μg SC | -8.06 | Negative |
| 31 | 91 | 5-C28 | BinJV/WNV_KUNproto_ 10μg SC | 1.70 | Negative |
| 32 | 91 | 5-C29 | BinJV/WNV_KUNproto_ 10μg SC | -35.71 | Negative |
| 33 | 91 | 5-C30 | BinJV/WNV_KUNproto_ 10μg SC | -32.95 | Negative |
| 34 | 91 | 5-C32 | BinJV/WNV_KUNproto_ 10μg SC | 11.82 | Negative |
| 35 | 91 | 5-C33 | BinJV/WNV_KUNproto_ 10μg SC | -5.42 | Negative |
| 36 | 91 | 5-C34 | BinJV/WNV_KUNproto_ 10μg SC | 18.06 | Negative |
| 37 | 91 | 5-C35 | BinJV/WNV_KUNproto_ 10μg SC | -25.89 | Negative |
| 38 | 91 | 5-C36 | BinJV/WNV_KUNproto_ 10μg SC | -3.28 | Negative |
| 39 | 91 | 5-C37 | BinJV/WNV_KUNproto_ 10μg SC | -34.29 | Negative |
| 40 | 91 | 5-C38 | BinJV/WNV_KUNproto_ 10μg SC | -3.86 | Negative |
| 41 | 91 | 5-C39 | BinJV/WNV_KUNproto_ 10μg SC | 9.74 | Negative |
| 42 | 91 | 5-C40 | BinJV/WNV_KUNproto_ 10μg SC | -12.62 | Negative |
| 43 | 91 | 5-E32 | BinJV/WNV_KUNproto_ 10μg SC | -22.99 | Negative |
| 44 | 90 | 5-D19 | BinJV/WNV_KUN2011_ 2μg IM | -9.88 | Negative |
| 45 | 90 | 5-D20 | BinJV/WNV_KUN2011_ 2μg IM | -2.28 | Negative |
| 46 | 90 | 5-D21 | BinJV/WNV_KUN2011_ 2μg IM | 9.80 | Negative |
| 47 | 90 | 5-D22 | BinJV/WNV_KUN2011_ 2μg IM | 4.84 | Negative |
| 48 | 90 | 5-D23 | BinJV/WNV_KUN2011_ 2μg IM | -13.54 | Negative |
| 49 | 90 | 5-D24 | BinJV/WNV_KUN2011_ 2μg IM | 9.36 | Negative |
| 50 | 90 | 5-D25 | BinJV/WNV_KUN2011_ 2μg IM | -3.62 | Negative |
| 51 | 90 | 5-D26 | BinJV/WNV_KUN2011_ 2μg IM | 23.77 | Negative |
| 52 | 90 | 5-D27 | BinJV/WNV_KUN2011_ 2μg IM | -5.62 | Negative |
| 53 | 90 | 5-D28 | BinJV/WNV_KUN2011_ 2μg IM | -5.22 | Negative |
| 54 | 90 | 5-D29 | BinJV/WNV_KUN2011_ 2μg IM | 10.39 | Negative |
| 55 | 90 | 5-D30 | BinJV/WNV_KUN2011_ 2μg IM | 6.60 | Negative |
| 56 | 90 | 5-D31 | BinJV/WNV_KUN2011_ 2μg IM | 9.89 | Negative |
| 57 | 90 | 5-D32 | BinJV/WNV_KUN2011_ 2μg IM | 7.21 | Negative |
| 58 | 97 | 5-D33 | BinJV/WNV_KUNproto_ 10μg IM | 1.66 | Negative |
| 59 | 97 | 5-D34 | BinJV/WNV_KUNproto_ 10μg IM | 4.11 | Negative |
| 60 | 97 | 5-D35 | BinJV/WNV_KUNproto_ 10μg IM | 8.94 | Negative |
| 61 | 97 | 5-D36 | BinJV/WNV_KUNproto_ 10μg IM | 9.59 | Negative |
| 62 | 97 | 5-D37 | BinJV/WNV_KUNproto_ 10μg IM | 8.40 | Negative |
| 63 | 97 | 5-D38 | BinJV/WNV_KUNproto_ 10μg IM | -5.50 | Negative |
| 64 | 97 | 5-D39 | BinJV/WNV_KUNproto_ 10μg IM | -11.93 | Negative |
| 65 | 97 | 5-D41 | BinJV/WNV_KUNproto_ 10μg IM | -7.29 | Negative |
| 66 | 97 | 5-D42 | BinJV/WNV_KUNproto_ 10μg IM | -5.31 | Negative |
| 67 | 97 | 5-D43 | BinJV/WNV_KUNproto_ 10μg IM | 10.57 | Negative |
| 68 | 97 | 5-D45 | BinJV/WNV_KUNproto_ 10μg IM | -14.19 | Negative |
| 69 | 96 | 5-D47 | BinJV/WNV_KUN2011_ 10μg IM | -0.49 | Negative |
| 70 | 96 | 5-D48 | BinJV/WNV_KUN2011_ 10μg IM | 6.95 | Negative |
| 71 | 96 | 5-D49 | BinJV/WNV_KUN2011_ 10μg IM | 0.55 | Negative |
| 72 | 96 | 5-D50 | BinJV/WNV_KUN2011_ 10μg IM | 10.70 | Negative |
| 73 | 96 | 5-E01 | BinJV/WNV_KUN2011_ 10μg IM | -17.35 | Negative |
| 74 | 96 | 5-E03 | BinJV/WNV_KUN2011_ 10μg IM | -16.05 | Negative |
| 75 | 96 | 5-E04 | BinJV/WNV_KUN2011_ 10μg IM | -1.06 | Negative |
| 76 | 96 | 5-E05 | BinJV/WNV_KUN2011_ 10μg IM | 5.90 | Negative |
| 77 | 96 | 5-E06 | BinJV/WNV_KUN2011_ 10μg IM | 13.95 | Negative |
| 78 | 96 | 5-E07 | BinJV/WNV_KUN2011_ 10μg IM | 2.44 | Negative |
| 79 | 96 | 5-E08 | BinJV/WNV_KUN2011_ 10μg IM | -27.77 | Negative |
| 80 | 96 | 5-E09 | BinJV/WNV_KUN2011_ 10μg IM | -20.24 | Negative |
| 81 | 96 | 5-E10 | BinJV/WNV_KUN2011_ 10μg IM | -15.52 | Negative |
| 82 | 96 | 5-E29 | BinJV/WNV_KUN2011_ 10μg IM | -5.92 | Negative |
| 83 | 102 | 5-E11 | BinJV/WNV_KUN2011_ 10μg IM | -5.84 | Negative |
| 84 | 102 | 5-E12 | BinJV/WNV_KUN2011_ 10μg IM | -3.37 | Negative |
| 85 | 102 | 5-E13 | BinJV/WNV_KUN2011_ 10μg IM | -7.63 | Negative |
| 86 | 102 | 5-E14 | BinJV/WNV_KUN2011_ 10μg IM | -2.43 | Negative |
| 87 | 102 | 5-E15 | BinJV/WNV_KUN2011_ 10μg IM | 2.08 | Negative |
| 88 | 102 | 5-E17 | BinJV/WNV_KUN2011_ 10μg IM | 4.51 | Negative |
| 89 | 102 | 5-E19 | BinJV/WNV_KUN2011_ 10μg IM | -4.51 | Negative |
| 90 | 102 | 5-E20 | BinJV/WNV_KUN2011_ 10μg IM | -3.60 | Negative |
| 91 | 102 | 5-E21 | BinJV/WNV_KUN2011_ 10μg IM | 8.38 | Negative |
| 92 | 102 | 5-E22 | BinJV/WNV_KUN2011_ 10μg IM | 18.19 | Negative |
| 93 | 102 | 5-E23 | BinJV/WNV_KUN2011_ 10μg IM | 11.82 | Negative |
| 94 | 102 | 5-E28 | BinJV/WNV_KUN2011_ 10μg IM | 2.58 | Negative |
| 95 | 102 | 5-E48 | BinJV/WNV_KUN2011_ 10μg IM | -5.88 | Negative |

**SUPPLEMENTARY FIGURES**


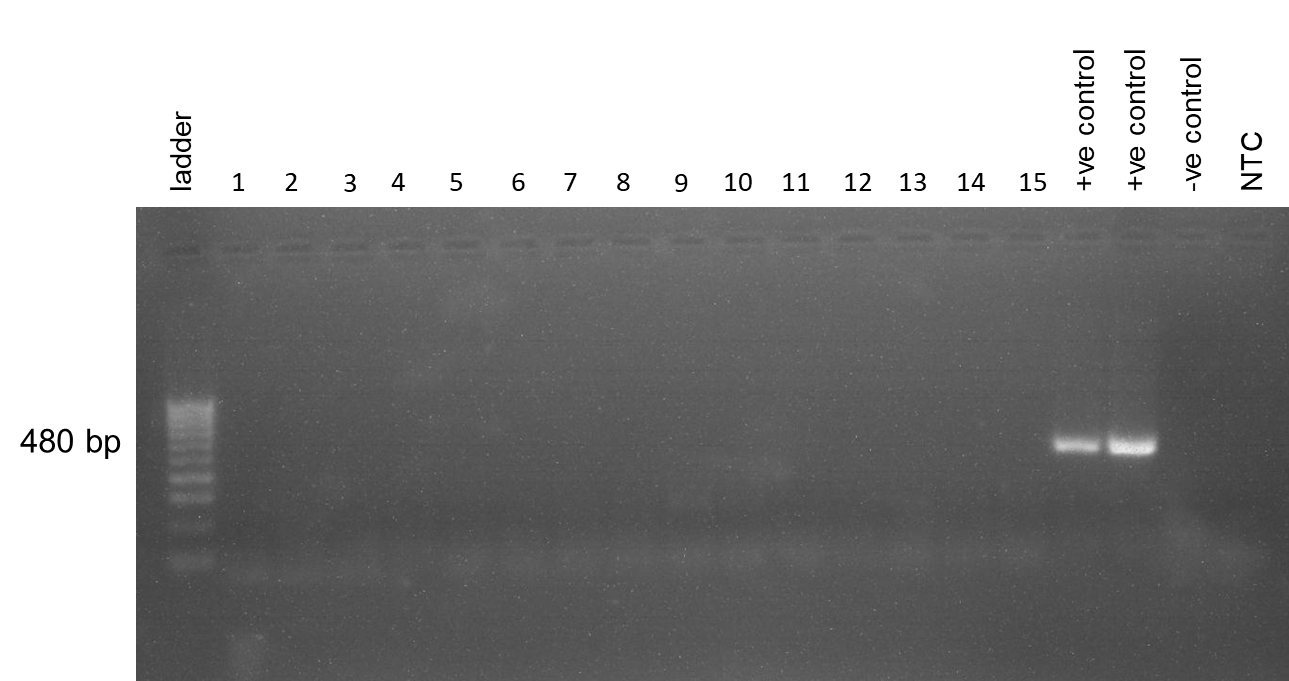


**Supplementary Figure 1. BinJV-NS5 specific RT-PCR on crocodile blood samples.** Lane 1. ladder, lanes 2-16: blood samples from vaccinated crocodiles, lanes 17-18: positive control, lane 19: negative control, lane 20: no template control.


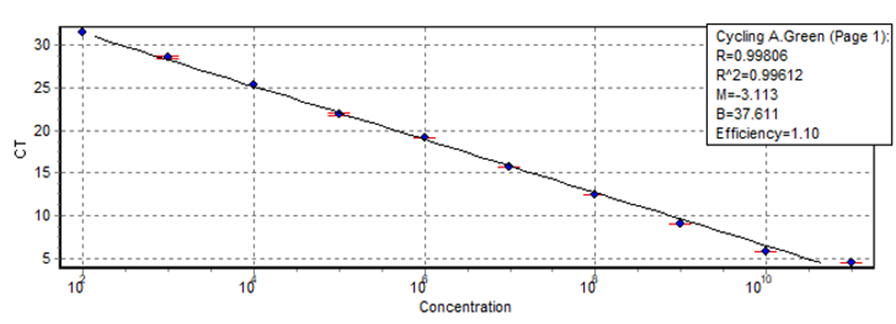


**Supplementary Figure 2. Standard curve to determine WNV_KUN_ infectious units equivalents from plasma qRT-PCR CT scores**. Ten-fold dilutions of WNV_KUN_ (10-1 to 10-7) were simultaneously assessed for infectious titre by TCID_50_ assay and levels of viral RNA by Taqman qRT-PCR. An exponential trend line was generated from the derived CT scores and calculated infectious units of the standard dilution series using the Excel Growth Function. Infectious unit equivalents were then predicted for each plasma sample from their derived CT scores. R2 value indicates line of best fit (closest to 1). The limit of detection was 6 - 10 TCID_50_/mL.


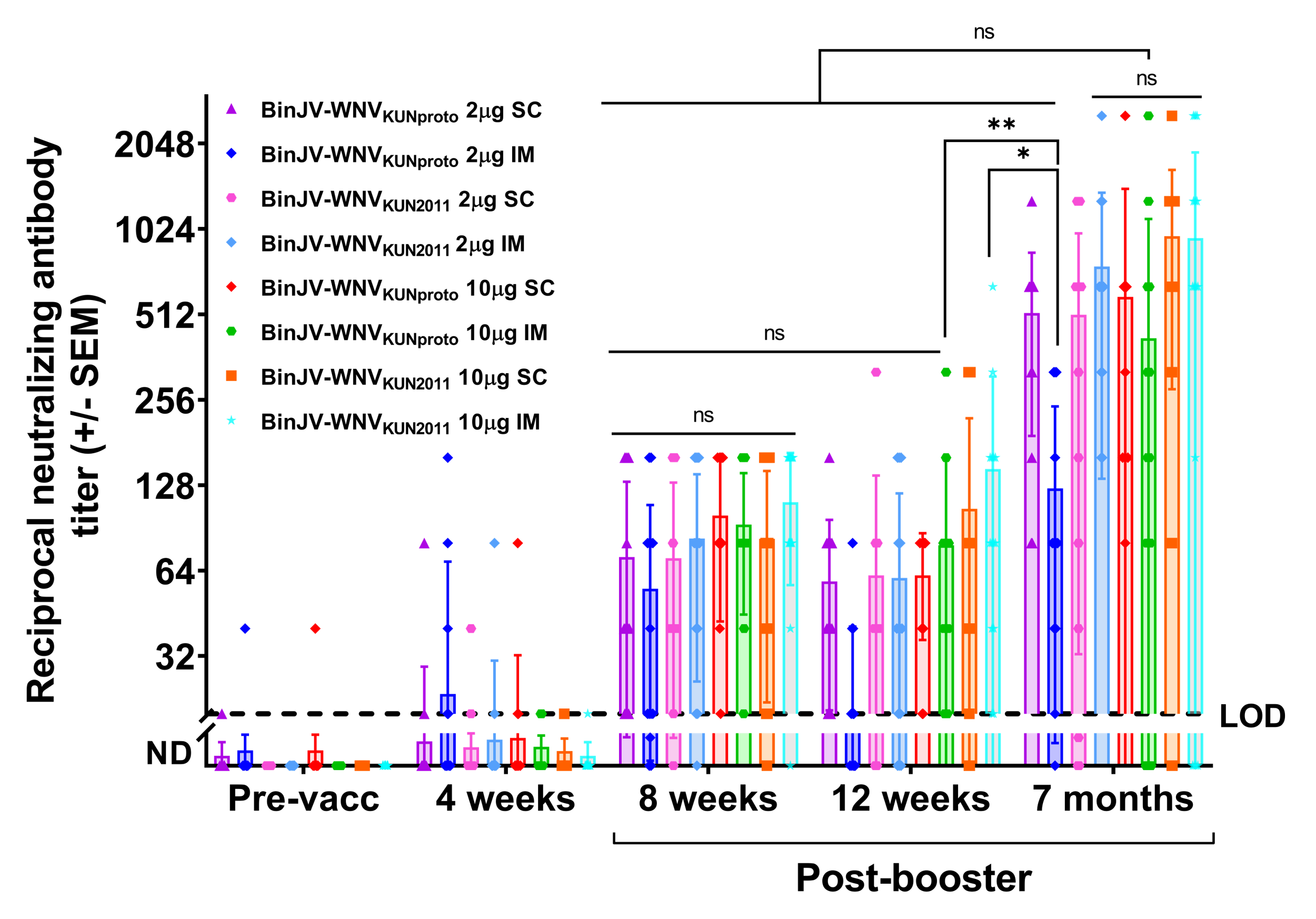


**Supplementary Figure 3.** Comparison of BinJV/WNV_KUN2011_ and BinJV/WNV_KUNproto_ vaccines vaccine response in vaccinated saltwater crocodiles. The dotted line represents the lower limit of detection (titre of 20) of virus-neutralising antibodies by VNT. The upper limit of detection of the assay was 2560. The data were subjected to two-way ANOVA with a Tukey’s post-test statistical analysis to test differences in virus neutralising antibody titres between treatment groups at various timepoints. Significant statistical difference thresholds are **p* ≤ 0.05, ***p* ≤ 0.01, ****p* ≤ 0.001.

**Supplementary Table 8. Sero-conversion rate and median neutralising antibody titres induced by BinJV/WNV_KUNproto_**

| **Timepoint** | **BinJV/WNV_KUNproto_ 2μg SC** | | | | **BinJV/WNV_KUNproto_ 10μg SC** | | | | **BinJV/WNV_KUNproto_ 2μg IM** | | | | **BinJV/WNV_KUNproto_ 10μg IM** | | | |
| --- | --- | --- | --- | --- | --- | --- | --- | --- | --- | --- | --- | --- | --- | --- | --- | --- |
|  | **Seroc. rate (%)** | **Median** | **UL** | **LL** | **Seroc. rate (%)** | **Median** | **UL** | **LL** | **Seroc. rate (%)** | **Median** | **UL** | **LL** | **Seroc. rate (%)** | **Median** | **UL** | **LL** |
| day 0 | 0 | <20 | 20 | <20 | 7.1 | <20 | 40 | <20 | 14.2 | <20 | 40 | <20 | 0 | <20 | <20 | <20 |
| 4 weeks | 14.2 | <20 | 80 | <20 | 28.6 | <20 | 80 | <20 | 35.7 | <20 | 160 | <20 | 21.4 | <20 | 20 | <20 |
| 8 weeks | 100 | 40 | 160 | 20 | 100 | 80 | 160 | 20 | 100 | 30 | 160 | <20 | 100 | 80 | 160 | 20 |
| 12 weeks | 100 | 40 | 160 | 20 | 100 | 80 | 80 | 20 | 78.5 | 10 | 80 | <20 | 100 | 60 | 320 | 20 |
| 7 months | 100 | 640 | 1280 | 80 | 100 | 240 | 2560 | 80 | 85.7 | 80 | 320 | <20 | 100 | 160 | 2560 | <20 |

**Supplementary Table 9. Sero-conversion rate and median neutralising antibody titres induced by BinJV/WNV_KUN2011_**

| **Timepoint** | **BinJV/WNV_KUN2011_ 2μg SC** | | | | **BinJV/WNV_KUN2011_ 10μg SC** | | | | **BinJV/WNV_KUN2011_ 2μg IM** | | | | **BinJV/WNV_KUN2011_ 10μg IM** | | | |
| --- | --- | --- | --- | --- | --- | --- | --- | --- | --- | --- | --- | --- | --- | --- | --- | --- |
|  | **Seroc. rate (%)** | **Median** | **UL** | **LL** | **Seroc. rate (%)** | **Median** | **UL** | **LL** | **Seroc. rate (%)** | **Median** | **UL** | **LL** | **Seroc. rate (%)** | **Median** | **UL** | **LL** |
| day 0 | 0 | <20 | < 20 | <20 | 0 | <20 | <20 | <20 | 0 | <20 | <20 | <20 | 0 | <20 | <20 | <20 |
| 4 weeks | 14.2 | <20 | 40 | <20 | 14.2 | <20 | 20 | <20 | 21.4 | <20 | 80 | <20 | 7.1 | <20 | 20 | <20 |
| 8 weeks | 92.8 | 40 | 160 | <20 | 100 | 80 | 160 | <20 | 92.8 | 80 | 160 | <20 | 92.8 | 120 | 160 | <20 |
| 12 weeks | 85.7 | 40 | 320 | <20 | 92.8 | 80 | 320 | <20 | 78.6 | 40 | 160 | <20 | 100 | 80 | 640 | 20 |
| 7 months | 85.7 | 640 | 1280 | <20 | 100 | 1280 | 2560 | <20 | 100 | 640 | 2560 | 160 | 92.8 | 640 | 2560 | <20 |

Seroc.: seroconversion

UL: upper limit

LL: lower limit


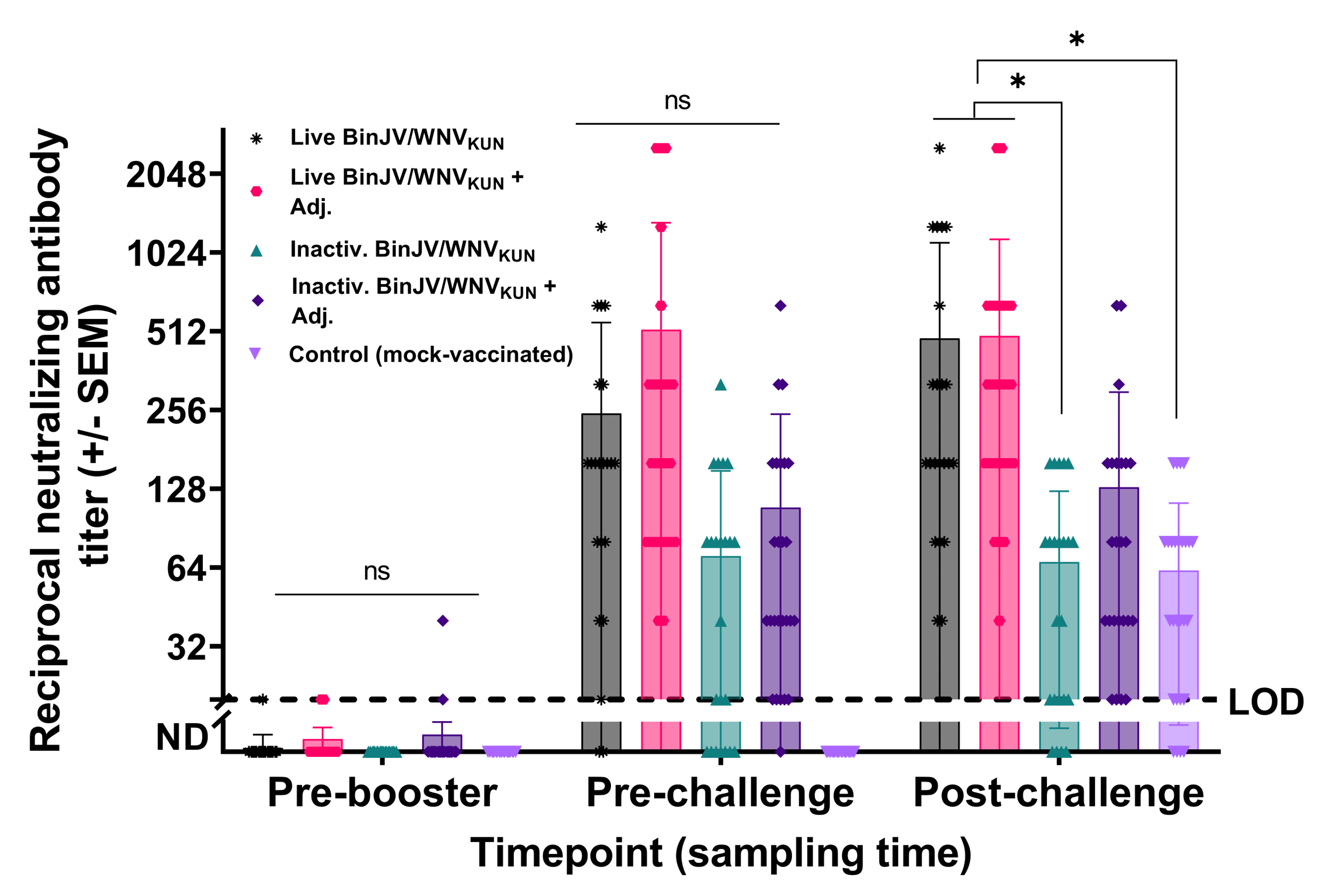


**Supplementary Figure 4.** Virus neutralisation antibody titres in saltwater crocodiles vaccinated with BinJV/WNV_KUN_ at multiple timepoints. The two-way ANOVA with a Tukey’s post-test statistical analysis to test differences in virus neutralising antibody titres between treatment groups at various timepoints. Significant statistical difference thresholds are **p* ≤ 0.05, ***p* ≤ 0.01, ****p* ≤ 0.001. Dotted line represents the limit of detection. The upper limit of detection of the assay was 2560.
